# Radiation-related superficial oral mucoceles: An under-recognized acute toxicity in head and neck cancer patients

**DOI:** 10.4317/medoral.22470

**Published:** 2018-09-28

**Authors:** Ana-Carolina Prado-Ribeiro, Alan-Roger Santos-Silva, Karina-Morais Faria, Wagner-Gomes Silva, Luciana-Estevam Simonato, Karina Moutinho, Thais-Bianca Brandão

**Affiliations:** 1 DDS, MSc, PhD, Dental Oncology Service, Instituto do Câncer do Estado de São Paulo [ICESP], Faculdade de Medicina da Universidade de São Paulo, São Paulo, Brazil; 2 DDS, MSc, PhD, Universidade Brasil, Campus Fernandópolis, São Paulo, Brazil; 3 DDS, MSc, PhD, Oral Diagnosis Department, Semiology Area, Piracicaba Dental School, University of Campinas (UNICAMP), Piracicaba, São Paulo, Brazil; 4 MD, Radiotherapy Service, Instituto do Câncer do Estado de São Paulo [ICESP], Faculdade de Medicina da Universidade de São Paulo, São Paulo, Brazil

## Abstract

**Background:**

Acute toxicity is usually defined as adverse changes occurring immediately or a short time after the start of oncological treatment.

**Material and Methods:**

Cross-sectional retrospective study performed with head and neck cancer patients who underwent radiotherapy from 2013 to 2016.

**Results:**

Ten (1.2%) patients developed SOMs during radiotherapy, most (80%) of which were men with a mean age of 59.5 years at diagnosis. SOMs mainly affected the floor of the mouth (60%) between the fourth and the sixth weeks of radiation therapy. All lesions were asymptomatic and spontaneously ruptured approximately 9 days after diagnosis.

**Conclusions:**

Although rare, SOMs may be regarded as an acute oral toxicity of head and neck radiotherapy.

** Key words:**Superficial oral mucoceles; head and neck cancer; radiation toxicity, acute toxicity.

## Introduction

Head and neck squamous cell carcinoma (HNSCC) is one of the leading causes of cancer-related morbidity and mortality, with approximately 500,000 new cases diagnosed yearly worldwide, most of which are recognized in advanced stages of disease progression. HNSCC treatment involves surgery, chemotherapy (CT), and head and neck radiotherapy (HNRT). More recently, concomitant HNRT and CT (chemoradiation, CRT) have significantly improved the 5-year survival rates of HNSCC patients. However, although effective in tumor control, CRT is associated with a myriad of debilitating toxicities to non-targeted tissues surrounding the tumor and a consequent reduction in the quality of life of cancer survivors (1,2).

Acute toxicity is usually defined as adverse changes occurring immediately or a short time after the start of medical treatment. In the case of HNRT, acute oral toxicities include mucositis, hyposalivation, dysgeusia, dysphagia and opportunistic infection mainly caused by *Candida albicans* and Herpes simplex virus. Oral chronic toxicity, in turn, can be defined as an adverse event occurring 3 months after the conclusion of HNRT that might persist over a long period of time (even for the rest of the patient’s life). The more relevant oral chronic toxicities secondary to HNRT include hyposalivation, trismus, radiation-related caries, osteoradionecrosis (ORN) and dysphagia, which eventually form a cluster of oral symptoms with the potential to impair overall oral function and also to negatively impact on patients’ quality of life (1,3,4).

A complete understanding of head and neck complications following cancer therapies is paramount to effectively characterize the burden of HNRT-related toxicities. In this scenario, underestimating oral toxicities may result in inappropriate care for HNSCC patients. Remarkably, there is still a lack of information regarding oral toxicities that might occur during cancer therapy in HNSCC patients (3).

Of note, conventional mucoceles were previously described in different anatomic sites of patients who concluded HNRT, including the maxillary sinus (5,6); the sphenoidal sinus (7-10); and the ethmoidal sinus (10,11). Therefore, the aim of this study was to describe an original case series of HNSCC patients who developed superficial oral mucoceles (SOM) during HNRT.

## Material and Methods

This was a cross-sectional retrospective study based on HNSCC patients submitted to HNRT at a single institution, from January 2013 to October 2016. The research protocol was approved by the Ethics Committee of the University of Sao Paulo Medical School (number 882.731).

All patients included in this study received dental treatment and oral hygiene instructions before the start of HNRT. Information regarding oral toxicities during cancer therapy was recorded following the institutional protocol of daily systematic oral examinations during the course of HNRT. The electronic charts of the patients were retrieved for clinical and epidemiologic characterization, including patient’s age, gender, tumor location, and cancer treatment protocols, among others.

## Results

Ten (1.2%) out of 834 HNSCC patients evaluated during the study period developed SOMs. None of the patients presented SOMs or any other soft tissue oral lesions before the start of HNRT. Eight (80%) patients were male and 2 (20%) female, with a mean age of 59.5 years at diagnosis of SOM. All patients presented an advanced clinical stage of disease (III/IV) at cancer diagnosis and a marked history of tobacco (100%) and alcohol (100%) consumption.

All selected patients were treated with 3D conformal tri-dimensional radiotherapy in 6mV linear accelerators on Synergy Platform (Elekta AB, Stockholm, Sweden), with radiation doses ranging from 60 to 70 Gy. Five (50%) patients [primary tumors were located in the oropharynx (20%), buccal mucosa (10%), palate (10%) and base of the tongue 1 (10%)] were treated with induction chemotherapy (paclitaxel+cisplatin) in combination with concurrent CRT (cisplatin), 2 (20%) oropharynx squamous cell carcinoma patients were treated with isolated RT, 1 (10%) patient (floor of the mouth squamous cell carcinoma) was treated by the combination of induction chemotherapy (paclitaxel+cisplatin) and RT, 1 (10%) patient (retromolar squamous cell carcinoma) was treated by surgery and adjuvant RT and 1 (10%) patient (larynx squamous cell carcinomas) was managed with surgery and adjuvant CRT (cisplatin).

Four (40%) patients developed SOM in the fourth week of radiation therapy, 1 (10%) in the fifth week, 2 (20%) in the sixth week, and 3 (30%) in the seventh week of HNRT. SOM lesions were predominantly observed in the floor of the mouth (60%) and none of the patients reported pain associated with the lesions. At diagnosis, SOM vesicles measured 0.7 cm in diameter, ranging from 0.3 a 3 cm. All SOM lesions spontaneously ruptured and regressed after a mean time of 9 days following onset (clinical diagnosis), ranging from 7 to 21 days. Although asymptomatic, 1 (10%) patient reported discreet and self-limiting pain (after 7 days) following the spontaneous rupture of the lesion associated with ulceration.

**Figure 1:**
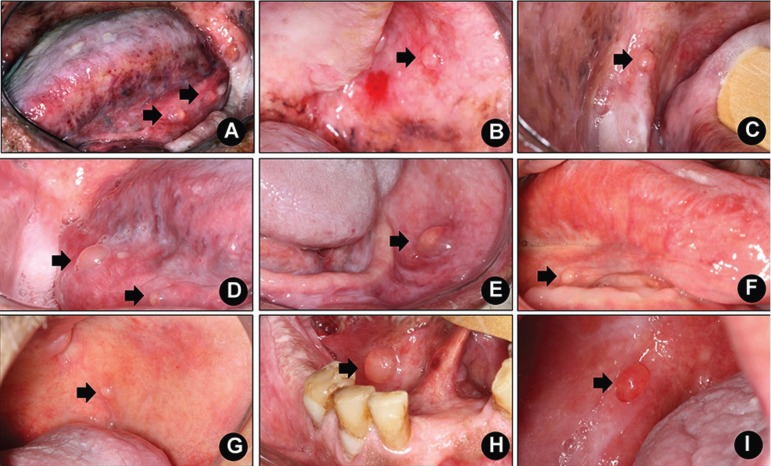
A, B and C. Multiple superficial oral mucoceles (arrows) affecting floor of the mouth, buccal mucosa and retromolar area (patient# 1). D. Two synchronous superficial oral mucoceles (arrows) on the right floor of the mouth (patient# 2). E. Superficial oral mucocele (arrow) on the left buccal mucosa (patient# 5). F. Superficial oral mucocele (arrow) affecting the right posterior floor of the mouth (patient# 6). G. Superficial oral mucocele (arrow) on the left posterior buccal mucosa (patient# 7). H. Superficial oral mucocele (arrow) on the right anterior floor of the mouth (patient# 8). I. Superficial oral mucocele (arrow) on the right alveolar ridge (patient# 9).

**Figure 2:**
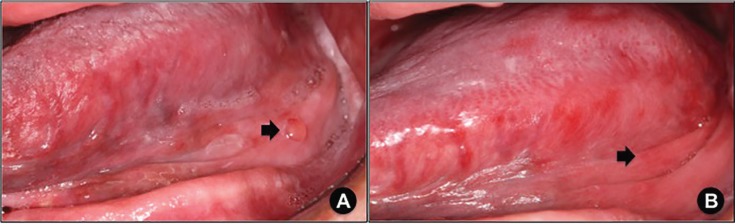
A. Superficial oral mucocele (arrow), which developed on the fifth week of radiotherapy, affecting the left posterior floor of the mouth (patient# 3). B. Seven-day-spontaneous rupture of superficial oral mucocele displayed on figure 2. A resulting in an erythematous background (arrow).

**Figure 3:**
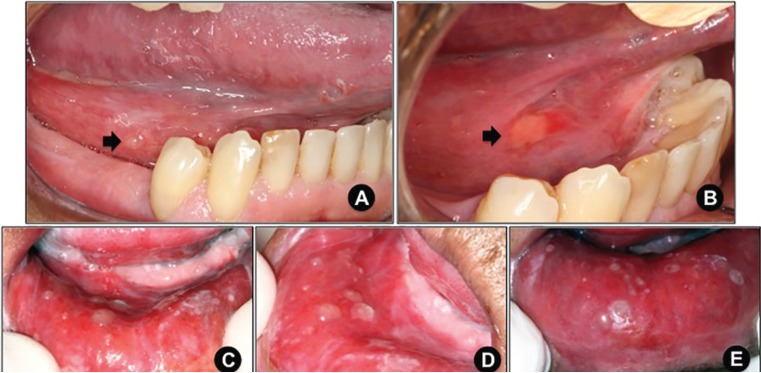
A. Superficial oral mucocele (arrow) on the right posterior floor of the mouth, which developed in the fourth week of radiotherapy (patient# 4). B. Seven-day-spontaneous rupture of superficial oral mucocele displayed on figure 3. A resulting in ulceration (arrow). C and D. Multiple superficial oral mucoceles developing on the lower lip in the seventh week of radiotherapy (patient# 9). E. A 3-week-follow-up image showing spontaneous partial rupture of lesions.

Detailed demographic and clinicopathological information concerning the patients included in this study are presented in  Table 1. Illustrative clinical images of the SOM cases are presented in Figures 1-3.

**Table 1:**
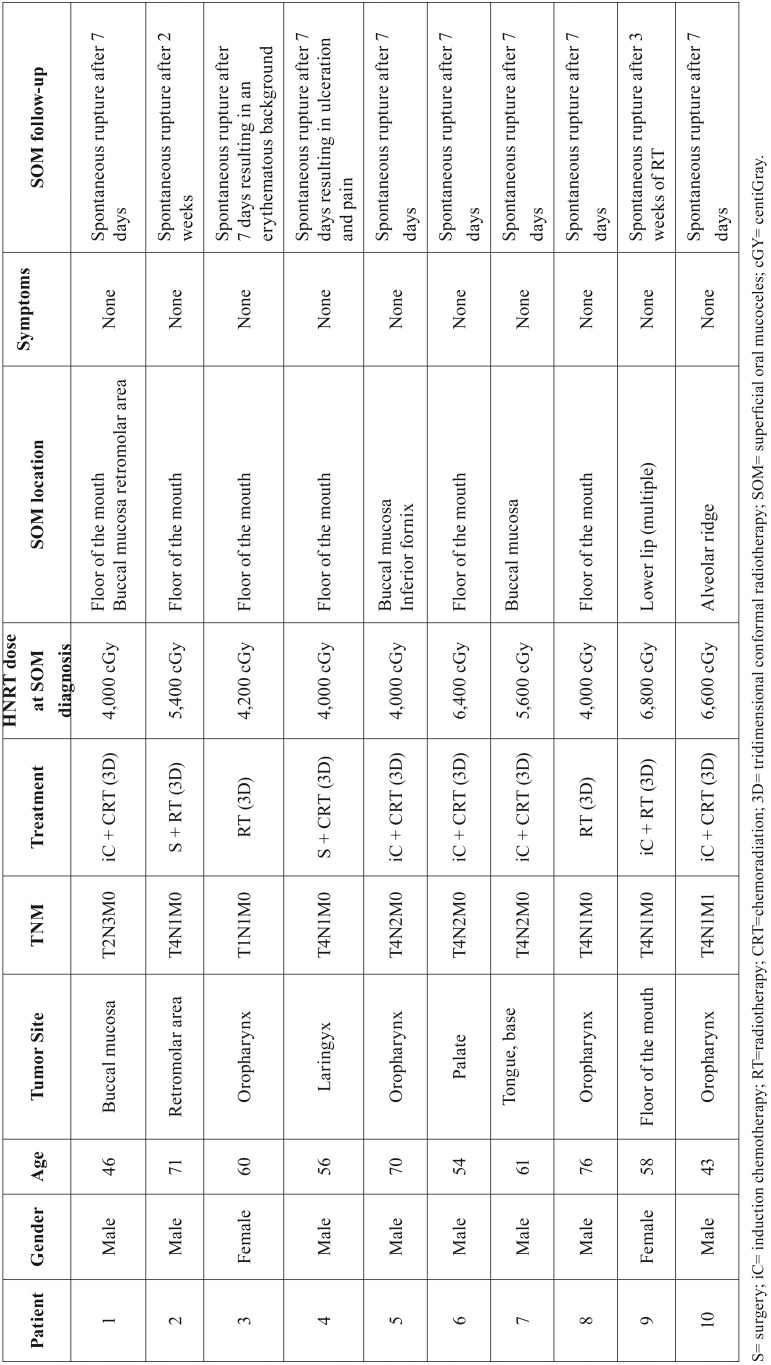
Clinical characteristics of head neck cancer patients diagnosed with radiation-related superficial oral mucoceles.

## Discussion

Eveson (12) originally described SOM in 1988 as small, translucent, subepithelial vesicles affecting the oral mucosa. In general, these lesions are more commonly observed in female patients over the age of 30 years old and most often affecting the soft palate and the buccal mucosae (13-15). Conversely, in the present case series, the majority of the patients were elderly males with most of the lesions affecting the floor of the mouth. The different demographic pattern of the present series was probably influenced by the fact that this is a series of SOM affecting HNSCC patients who are typically composed of elderly male populations (16). It is also relevant to mention that the patients included in this study were typical in terms of cancer demographic features, in the sense that most of them presented oral cavity and oral pharynx advanced squamous cell carcinomas, which were treated by HNRT, exclusively or combined with surgery or CT (17).

Recently, Keshet *et al.* (18) reported two cases of SOM in patients who concluded HNRT. In this previous report, both patients were female, and affected by advanced tongue squamous cell carcinoma. One of the patients presented three synchronous SOMs affecting the buccal and labial mucosa one month after the conclusion of isolated HNRT (6,500 cGy). The other patient presented multiple SOMs in the soft palate three months after the conclusion of adjuvant HNRT (total dose 6,400 cGy). In contrast to their results, herein we suggest that SOM may also develop as an acute oral toxicity of HNRT since the majority of the patients studied developed SOM between the fourth and sixth weeks of radiation therapy (with cumulative doses ranging from 4,000 cGy to 6,800 cGy). Possibly, this is the first evidence of SOMs during the course of HNRT, which were recognized early because all patients included in this study were submitted to systematic oral evaluations throughout the course of HNRT, which is part of the oral care treatment protocols of our service.

SOM can present as single or multiple mucosae vesicles and often present as “short-lived lesions” because they frequently rupture and cause superficial painful ulcers (5,6,8). In the current case series, none of the patients reported SOM-associated pain and they were all unaware of the existence of these lesions. Most patients presented small single SOM lesions, all of which ruptured spontaneously in approximately 9 days, leading to a prompt healing process. Of note was one patient in whom the spontaneous rupture resulted in a discreet and transitory painful sensation and another patient who presented multiple SOMs that persisted for 3 weeks after the conclusion of HNRT.

A recent study performed by Treister *et al.* (19) prospectively evaluated 458 patients with oral chronic graft-versus-host disease (cGVHD) aiming to characterize the clinical features of oral manifestations of cGVHD. Interesting, they reported a high incidence of mucoceles (43%) in patients with oral cGVHD and dryness. When taken together, these results suggest that mucoceles and SOM might be regarded as relevant in the context of cancer treatment-related oral lesions.

In terms of etiopathogenesis, Jensen (20) suggested that the increased pressure caused by mucous plugs in the intraepithelial squamous-cell-lined portions of the minor salivary glands ducts could cause duct rupture and lead to the development of SOM. This original evidence that SOMs may be an early event in HNRT validates the above-mentioned hypothesis because it is well-known that ionizing radiation causes permanent damage to the major and minor salivary glands, reducing the salivary flow rate and changing the qualitative composition of saliva; this initially becomes evident during the first week of HNRT and is clinically detectable by the presence of a highly viscous and mucous saliva (21-23). Hence, HNRT-related SOM may develop as a consequence of the increased viscosity of saliva, which increases the pressure in the ducts of minor salivary glands, causing duct rupture and sub-epithelial saliva accumulation.

Although the current study seems to be original in describing the frequency and consistency of SOM in patients with HNSCC who underwent radiotherapy, it has limitations that should be considered in further clinical studies, such as its retrospective nature, the limited number of patients and examiners.

In conclusion, SOM should be considered an unrecognized acute oral toxicity of HNRT. Future clinical studies are necessary to confirm the apparent self-limiting and non-painful behavior of these lesions and also to access the potential of SOM to rupture during HNRT and negatively impact the evolution of oral mucositis.
